# Tissue clearing and immunostaining to visualize the spatial organization of vasculature and tumor cells in mouse liver

**DOI:** 10.3389/fonc.2023.1062926

**Published:** 2023-03-28

**Authors:** Nicola Frenkel, Susanna Poghosyan, Jan Willem van Wijnbergen, Lotte van den Bent, Liza Wijler, André Verheem, Inne Borel Rinkes, Onno Kranenburg, Jeroen Hagendoorn

**Affiliations:** Laboratory for Translational Oncology, University Medical Center Utrecht, Utrecht, The Netherlands

**Keywords:** tissue clearing, liver vasculature, CLARITY, 3DISCO, iDISCO, tumor micro environment, colorectal cancer, liver metastasis

## Abstract

The liver has a complex and hierarchical segmental organization of arteries, portal veins, hepatic veins and lymphatic vessels. In-depth imaging of liver vasculature and malignancies could improve knowledge on tumor micro-environment, local tumor growth, invasion, as well as metastasis. Non-invasive imaging techniques such as computed tomography (CT), magnetic resonance imaging (MRI) and positron-emission transmission (PET) are routine for clinical imaging, but show inadequate resolution at cellular and subcellular level. In recent years, tissue clearing – a technique rendering tissues optically transparent allowing enhanced microscopy imaging – has made great advances. While mainly used in the neurobiology field, recently more studies have used clearing techniques for imaging other organ systems as well as tumor tissues. In this study, our aim was to develop a reproducible tissue clearing and immunostaining model for visualizing intrahepatic blood microvasculature and tumor cells in murine colorectal liver metastases. CLARITY and 3DISCO/iDISCO+ are two established clearing methods that have been shown to be compatible with immunolabelling, most often in neurobiology research. In this study, CLARITY unfortunately resulted in damaged tissue integrity of the murine liver lobes and no specific immunostaining. Using the 3DISCO/iDISCO+ method, liver samples were successfully rendered optically transparent. After which, successful immunostaining of the intrahepatic microvasculature using panendothelial cell antigen MECA-32 and colorectal cancer cells using epithelial cell adhesion molecule (EpCAM) was established. This approach for tumor micro-environment tissue clearing would be especially valuable for allowing visualization of spatial heterogeneity and complex interactions of tumor cells and their environment in future studies.

## Introduction

The liver consists of a complex and hierarchical segmental organization of arteries, portal veins, hepatic veins, and lymphatic vessels. These are also a crucial component of the liver-tumor micro-environment and play an important role in processes such as local tumor growth, invasion, as well as onward metastasis. For visualization of the three-dimensional (3D) liver vessel organization and malignancies, imaging techniques such as computed tomography (CT), magnetic resonance imaging (MRI) and positron-emission transmission (PET) are used clinically. However, for fundamental and translational research, these imaging techniques lack the appropriate cellular resolution for three-dimensional imaging at tissue level (1, 2).

**Figure 1:**
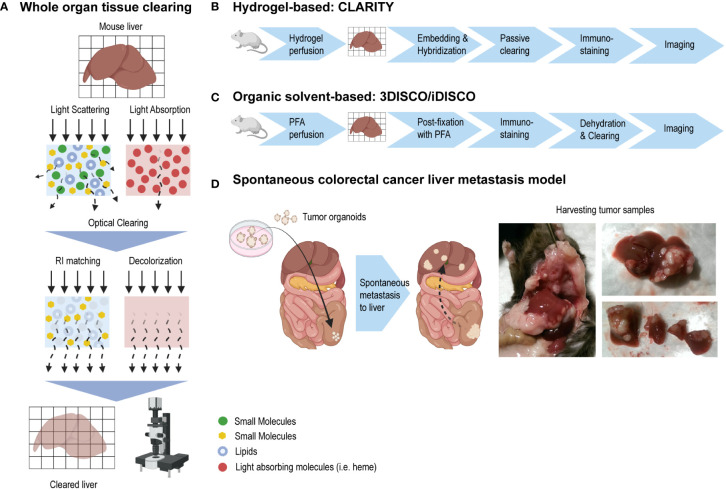
Whole organ tissue clearing using CLARITY and 3DISCO/iDISCO methods. **(A)** A tissue’s opacity is mainly caused by light scattering (due to the heterogeneity of the tissue components optical characteristics or refractive indexes) and light-absorbing compounds such as heme. Light scattering and light absorption hinder the light as it travels through a tissue. By using tissue clearing techniques, lipids are extracted and refractive indexes are better matched. Steps to remove light absorbing compounds are taken. The result is a “transparent” tissue in which microscopy imaging depth has greatly been improved. **(B)** CLARITY is a tissue clearing method which uses a tissue-gel hybrid to support and maintain the tissue’s architecture. **(C)** 3DISCO and iDISCO methods do not make use of a hydrogel scaffold like CLARITY, but use organic solvents instead for optical tissue clearing. **(D)** In a murine model, colorectal cancer organoids were implanted into the mouse cecum, after which the tumor cells spontaneously metastasized to the liver. Liver tumor samples were harvested and used to establish a tumor tissue clearing protocol.

Traditionally, histological techniques such as immunohistochemistry can provide excellent cellular and subcellular resolution. However, they require thin tissue sections ranging in thickness between 3 and 5 µm due to imaging depth constraints. 3D renderings can be constructed by means of serial sectioning and post-image reconstruction, a labor intensive and therefore inefficient process. Moreover, sectioning causes tissue damage, distortion and artifacts (1–6). Therefore, tissue clearing, an approach clarifying tissues by reducing light scattering and absorption hereby improving high resolution imaging depth, provides a promising alternative to visualize liver vasculature and malignancies. The first attempts at tissue clearing were performed over 100 years ago (7, 8). Since then, various tissue clearing methods have been devised, adjusted, and optimized. Clearing agents improve the trajectory of light through tissues by eliminating certain biomolecules such as lipids and light-absorbing pigments, hereby homogenizing the refractive index of the tissue, rendering the tissue “transparent” (1–6). (**Figure 1A**) The ideal method should yield excellent clearing while also maintaining native tissue architecture, preserve endogenous fluorescent proteins, and allowing specific immunolabeling. Generally, three groups of current clearing methods exist: 1) Organic-solvent based (such as 3DISCO, iDISCO, BABB) 2) aqueous-based (such as CLEAR, Scale, SeeDB, CUBIC, FRUIT), and 3) hydrogel embedding (such as CLARITY, PACT, PARS). Compared to thin tissue sections of only several micrometres used for histological techniques, successful clearing and imaging has been established for thick tissue sections and even whole mouse organs, reaching excellent imaging depths of several millimetres (1–6).

While some tissue clearing methods have been shown to render liver tissue reasonably optically transparent, liver tissue clearing is generally hampered by its high content of light-absorbing heme (9–15). Moreover, optimal tissue clearing involves the use of strong organic solvents or detergents which affect native biomolecules and tissue architecture to the point of them being severely altered, damaged or lost (2, 3, 6, 16). Here, our aim was to develop a robust and reproducible murine tissue clearing and immunostaining model for the visualization of intrahepatic blood microvasculature, as well as tumor cells in murine colorectal cancer (CRC) metastases. As the clearing methods CLARITY and 3DISCO/iDISCO+ are well established for compatibility with immunolabelling, albeit mainly in the neurobiology field, these methods were investigated for clearing and immunolabelling liver tissue and CRC metastasis samples. We showed that the hydrogel-based CLARITY with passive clearing method in liver tissue resulted in tissue integrity unsuitable for immunolabelling. Using 3DISCO/iDISCO+, we could successfully render liver and CRC tumor samples optically transparent and immunolabel the intrahepatic endothelial system as well as CRC metastases, making this approach a suitable technique for future studies of intrahepatic vasculature as well as the spatial heterogeneity and interactions of tumor cells and their environment in future studies.

## Materials and methods

### Tumor organoid culture

From a previously described transgenic mouse model with conditional activation of the Notch1 receptor as well as a p53 deletion in the digestive tract (17), organoids were derived from spontaneously formed colon tumors. All of the derived tumors showed mutations of either Ctnnb1 or Apc genes, implicating classical Wnt pathway activation (17). Organoids were FACS-sorted after being transduced using a lentiviral vector expressing luciferase and tdTomato (pUltra-Chili-Luc, Addgene #48688). Culturing was done at 37°C in a humidified atmosphere containing 5% CO2 using a 3D matrix structure (droplets of Growth Factor Reduced Basement Membrane Extract (BME; Amsbio) and Advanced DMEM/F12 (Thermo Fisher Scientific) medium which was supplemented with 1% Penicillin-Streptomycin (Gibco), 1% HEPES buffer, 2 mM Glutamax (Invitrogen), 2% B27 supplement (Invitrogen), 100 ng/ml Noggin (produced by lentiviral transfection), 10 nM murine recombinant FGF (PeproTech) and 1 mM n-Acetylcysteine (Sigma-Aldrich). Passaging was done weekly using TrypLE Express (Gibco) and medium was refreshed biweekly or earlier if needed based on organoid density.

### Animals

Male C57BL/6 (for non-tumor optical clearing experiments) and C57BL/6NCrl mice (for colorectal (CRC) tumor inoculation experiments), aged 8-10 weeks, were housed in groups of maximum 5 animals per cage in open cages with tissues and contact bedding. The animals were kept on a 12:12 h light (7AM)/dark (7PM) cycle at 20-24°C, 45-60% humidity and received water and AIN-93M pellets (Ssniff Spezialdiäten GmbH) ad libitum. Mice were acclimated for minimally 1 week before tumor inoculation. All mice were assessed biweekly for body weight and physical tumor progression. Bioluminescent imaging was performed *in vivo* weekly to monitor tumor progression and metastasis formation. Mice were anesthetized using isoflurane 4% for induction and 2% for maintenance, with 1.6 l/min oxygen. They received an intraperitoneal injection with 100 μL of D-luciferin (VivoGloTM Luciferin, Promega) in PBS. Mice were then imaged at 1s exposure/image for 5min, using the PhotonIMAGERTM RT system (Biospace Lab, Paris, France) an d M3 Vision software (Biospace Lab).

### *In vivo* surgical inoculation of colorectal cancer organoids

Mice were anesthetized using isoflurane and analgesic buprenorphine (0.1 mg/kg) was administered subcutaneously. Colorectal cancer organoids were dissociated using TrypLE (Gibco). A total of 250.000 single cells were embedded in 6μL of 75% Rat Tail Type I Collagen (Corning) droplets and 25% neutralization buffer; AlphaMEM powder (Life Technology), 1 M HEPES buffer pH 7.5 (Invitrogen) and NaHCO3 (Sigma). The organoids were left to recover overnight. The next day, the cecum was exposed by median laparotomy. A small 2mm incision was made into the cecum using a scalpel and bleeding was stemmed using cotton tips. The collagen drops containing 1-day old CRC organoids were shortly air-dried and gently pushed into the incision site, and afterwards sealed by placing Seprafilm (Genzyme) over the incision site. The peritoneum wall and skin were then sutured. Mice were monitored closely for 2 days to ensure proper incision closure. Upon signs of tumor growth and metastasis formation, determined clinically and by bioluminescence imaging, mice were sacrificed and samples were harvested as stated below.

### Tissue clearing method: CLARITY

**Figure 2:**
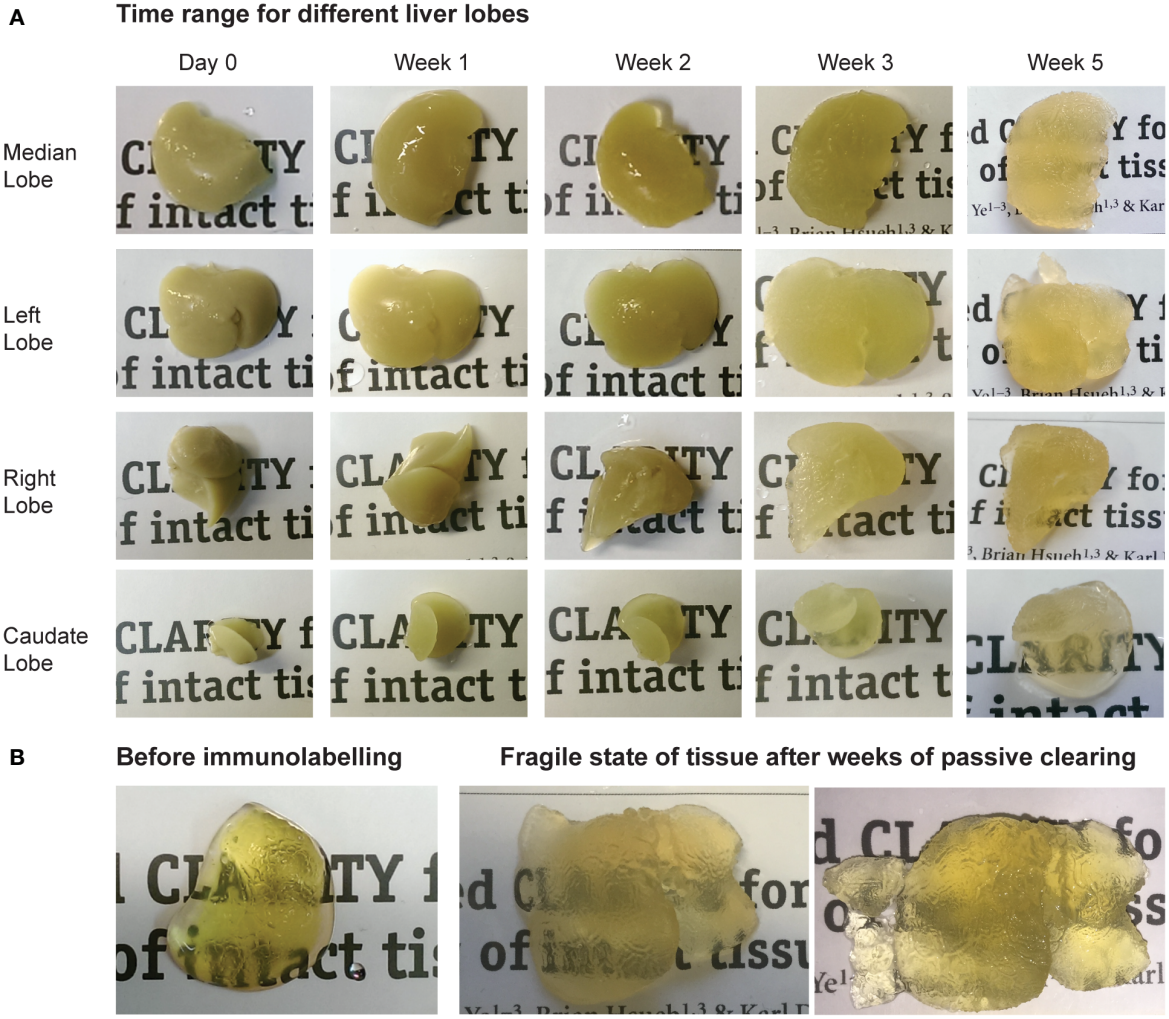
Tissue clearing using hydrogel-based CLARITY method. **(A)** Time range showing the passive clearing process for different liver lobes. **(B)** Before immunolabeling, adequate transparency of tissue was reached for the liver lobes. As passive clearing required several weeks of the tissues being in contact with strong detergent solutions before they were rendered adequately transparent for imaging, the samples became extremely fragile and would easily fragment during experimental handling.

The CLARITY method has been previously described (18). To form the hydrogel monomer (HM) solution, the following were combined cold and kept on ice: (in total volume 400ml) 4% acrylamide (BioRad), 1x TBS, 4% PFA, 0.25% VA-044 (FujiFilm), in 220ml dH2O. HM solution was stored in 50ml Falcon tubes at -20°C. On the day of the experiment, the HM solution was thawed on ice and gently mixed by inverting. A dedicated mouse surgery room was used. All operations were performed during the afternoon. After anesthesia, mice were given Temgesic (0,3mg/ml, Indivior Europe Limited) by subcutaneous injection preoperatively at 0.05 mg/kg. Median laparotomy was performed, the diaphragm was cut, the xiphoid was lifted and held in place with a clamp and the heart was exposed. A 23G needle was inserted into the left ventricle and the right atrium was opened with a small cut. Transcardial perfusion was performed using an infusion pump to infuse first 20ml ice-cold PBS and 20ml ice-cold HM solution immediately afterwards. Liver lobes were harvested and were placed in a 50ml Falcon tube containing ice-cold 30ml HM solution. Murine liver lobes differ in size (as seen in **Figure 2**). The liver lobe samples had a maximum length of 30mm and maximum width of 20mm, with a maximum thickness of 10mm. The liver lobes were incubated at 4°C for 12hrs. As preparation for tissue embedding, oxygen must be removed from sample tubes as oxygen inhibits hydrogel polymerization. A layer of mineral oil of 5mm thick was placed on top of the HM solution. The falcon tube was then placed in a 37°C water bath for 3-4 hrs. The liver lobes were extracted carefully from the solidified hydrogel and the hydrogel was removed from the sample surfaces using gentle rubbing motion with disposable Kimwipes (Kim Tech). Liver lobes samples were washed 2x for 24hrs in 50ml SDS/Boric Acid clearing solution (SBC) solution (stock solution: 20% SDS (Roche) and 1M boric acid buffer (Sigma) in H2O (pH adjusted using NaOH to pH 8.5). Final clearing solution was freshly prepared each time by diluting stock solution fivefold in H2O). The sample was then incubated at 37°C on a swivel platform in SBC solution for several weeks (for the complete liver lobes incubation time of 5-6 weeks was used). Fresh clearing solution was added 2x a week. Afterwards, samples were washed 2x with PBST (PBS + 0.1% Triton X-100 (Sigma)) at 37°C on a swivel platform for 24hrs each wash. Samples were incubated with primary antibodies in PBST for 7 days at 37°C on a swivel platform. To the Ab/PBST solution, 0.01% sodium azide (Merck) was added to prevent microbial growth. Samples were washed for 2-3 days in PBST buffer at 37°C on a swivel platform. Fresh buffer was added every 4-6hrs. Then samples were incubated with secondary antibodies and DAPI in PBST buffer for 2-3 days at 37°C on a swivel platform. Antibodies used were (at 1:50) Phalloidin-Actin Alexa 488 (A12379, Life Technology), Phalloidin-Actin Alexa 568 (A12380, Life Technology), CD31 (01951D Pharmingen, sc-1505 Santa Cruz), vWF, panendothelial marker MECA-32 (120502, Biolegend). Secondary antibodies (1:200): Goat anti-Rat Alexa 488, Donkey anti-Goat Alexa 488, Donkey anti-Sheep Alexa 647 (Invitrogen). DAPI (Biolegend) was used at 1:100. After immunolabelling, samples can be stored in PBST with 0.01% sodium azide at 4°C. For refractive index homogenization, before imaging, samples were submerged in glycerol for 24hrs in the dark at room temperature. Once the tissues are cleared, imaging with Ultramicroscope II (LaVision BioTec) lightsheet microscope or confocal microscopy (Zeiss LSM700) was performed. Imaging analysis was performed using Imaris software (Version 8.4, Bitplane).

### Tissue clearing method: 3DISCO/iDISCO+

**Figure 3:**
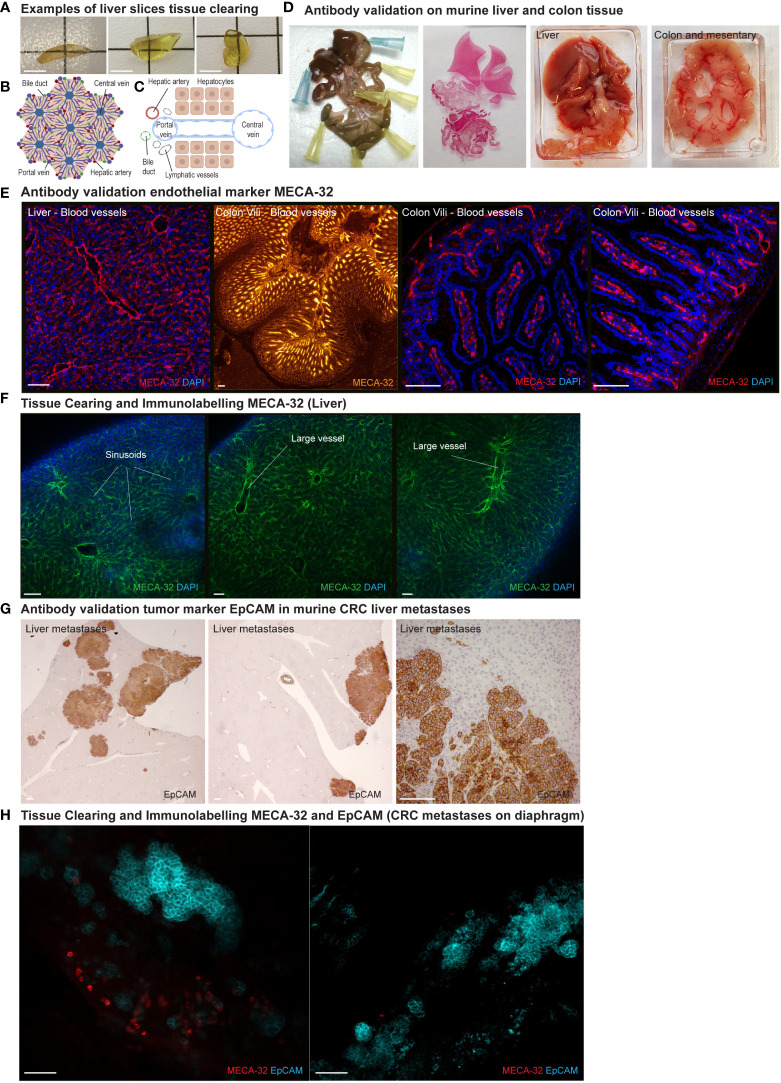
Tissue clearing: Vasculature and CRC tumor cells using 3DISCO/iDISCO. **(A)** Examples of transparency of liver samples after tissue clearing with the 3DISCO/iDISCO method. Scale bar = 5 mm **(B, C)** Structural organization of liver tissue and vasculature. **(D)** Tissue samples of murine liver and colon, used for antibody validation on tissue sections. **(E)** Antibody validation on liver and colon (vili) sections of pan-endothelial marker MECA-32 for blood vessel immunolabelling. DAPI (blue), MECA-32 (red and orange) Scale bar = 100μm **(F)**. Visualization of blood endothelial microvasculature of the liver using endothelial marker MECA-32 (green). (DAPI in blue). MECA-32 showed specific staining of the liver sinusoids and larger vessels. Scale bar = 100μm **(G)** Antibody validation of tumor marker EpCAM in murine CRC liver metastases. Scale bar = 100μm. **(H)** To use the model for tumor micro-environment imaging, a robust tumor marker is needed. EpCAM (turquoise) showed strong specific staining of tumor cells in a metastatic lesion on diaphragm tissue. MECA-32 (red) Scale bar = 100μm.

The 3DISCO/iDISCO+ method has been described previously (3, 19–21). Mouse transcardial perfusion and sample harvesting were performed as described above using ice-cold PBS and 4% PFA (Sigma). Liver lobes were extracted. The liver lobes had a maximum length of 30mm and maximum width of 20mm, with a maximum thickness of 10mm. The median, left and right lobes were cut into smaller samples with a maximum length of 20mm and maximum width of 10mm, maximum thickness of 10mm. The smaller caudate lobes were cut in half (maximum length 5mm x width 5mm, maximum thickness 3-4mm) and used for antibody validation (after antibodies had been tested in immunolabeling on immunohistochemistry (IHC) and frozen sections, see **Figures 3E, G**). Liver samples were incubated in 50ml Falcon tubes in ice-cold 4% PFA for 12hrs. The next day, samples were left for 1hr at RT on a roller bank and washed 3x with PBS for 30min. Samples were dehydrated using MeOH (Merck)/PBS: 50-80-100% MeOH, 1,5hr for each step at RT with agitation. Samples were then transferred to a solution of MeOH and 6% H2O2 (Merck) for bleaching and left to incubate overnight at 4°C, protected from light. Afterwards, samples were rehydrated in 100% MeOH (2x), 80% and 50% MeOH in PBS, 1,5hrs at RT with agitation for each step. Samples were then incubated in PBSGT (0.2% gelatin (Sigma, from porcine skin) and 0.5% Triton X-100 (Sigma) in PBS) at RT for 24hrs – 4 days depending on sample size. Samples were incubated with primary antibodies in PBSGT + 0.1% saponin (ThermoFisher) for 7 - 14 days depending on sample size at 37°C on swivel platform. Samples sized 20mm x 10mm were incubated for 14 days, while smaller samples (caudate lobes) sized 10mm x 10mm were incubated for 7 days. After washing 6x with PBSGT at RT with agitation, samples were incubated with secondary antibodies and DAPI in PBSGT and 0.1% saponin overnight – 2 days depending on sample size at 37°C on swivel platform. Smaller samples of 5x5mm were incubated overnight, while the larger samples of 20x10mm were incubated for 2 days. To avoid precipitates, secondary antibody solutions were passed through 0.22μm filter. Antibodies used (1:500): Epcam (SinoBiological, 50591-R002) and Panendothelial marker MECA-32 (120502, Biolegend). Secondary antibodies (1:500) were Goat anti-Rat Alexa 568 and Goat anti-rabbit Alexa 647 (Invitrogen). DAPI (Biolegend) was used at 1:100. Samples were washed 6x with PBSGT at RT with agitation. Samples could be stored at 4°C until clearing. Clearing step: 20-40-60-80-100% MeOH/PBS, 1 hr for each step at RT under agitation, protected from light. Then incubation for 3hrs – overnight in 2/3 DCM (ThermoFisher) + 1/3 MeOH, protected from light. Then samples were incubated in 100% DCM 30min and transferred to 100% DBE (Sigma) until cleared. Sample can be kept in DBE at RT protected from light, in glass vial. Once the tissues were cleared, imaging with light sheet microscopy (Ultramicroscope II, LaVision BioTec) or confocal microscopy (Zeiss LSM700) was immediately performed. When using the light sheet microscope, the imaging chamber was filled with DBE containing the sample. Due to tissue shrinkage inherent during this clearing protocol, final tissue thickness when used for imaging was a maximum of 5-6mm where an imaging depth of 5mm was reached. Imaging analysis was performed using Imaris software (Version 8.4, Bitplane).

### Ethical guidelines

This research was approved by the National Competent Authority (Licence number AVD115002016614), which is advised by the Animal Ethics Committee, and conducted in accordance with institutional guidelines for the care and use of laboratory animals as well as the Dutch Law on Animal Experiments and the European Directive 2010/63/EU.

## Results

### Tissue integrity hindering immunolabelling using hydrogel-based CLARITY

Using the hydrogel-based clearing method called CLARITY(20), tissue-gel hybrid samples of liver tissue were created. (**Figure 1B**) While liver samples could be made optically transparent, unfortunately the passive clearing took several weeks for obtaining adequate tissue transparency, which caused the tissues to become extremely fragile. (See **Figures 2A, B**) Samples were immunolabeled with Phalloidin-Actin and DAPI to evaluate immunolabeling, tissue structure and integrity after the CLARITY process. Unfortunately, this resulted in epitope damaging and non-specific labelling (data not shown). Because of the tissues’ fragile state, the samples were extremely difficult to handle and would fragment easily. Further rounds of adjusted experimental procedures could not improve either the immunolabeling or tissue integrity.

### Improved tissue integrity using organic-solvent based 3DISCO/iDISCO+

Different from CLARITY, this method does not make use of hydrogel embedding and hybridization, but uses organic solvents to clear tissues. (**Figure 1C**). Liver samples were cleared rapidly while maintaining tissue integrity. (**Figure 3A**).

### Immunolabelling intrahepatic vasculature using 3DISCO/iDISCO+

Liver tissue consists of remarkably organized and structured vasculature comprising bile ducts, portal veins, central veins, hepatic arteries, sinusoids and lymphatic vessels. (**Figures 3B, C**) To visualize intrahepatic endothelial vasculature, firstly CD31 (cluster of differentiation 31, also known as platelet endothelial cell adhesion molecule PECAM-1) was used. While sinusoids and larger vessels could be distinguished ( **Supplementary Figure 1A**), CD31 showed no specific staining as it strongly resembled controls using only the secondary antibody (data not shown). In contrast, immunostaining with MECA-32 (a panendothelial cell antigen on murine endothelium) showed specific labelling of the liver sinusoidal and larger endothelial structures. (**Figure 3F**) Before usage in the clearing and immunolabelling protocol, immunofluorescent MECA-32 blood vasculature labelling was validated on liver and colon tissue sections. (**Figures 3D, E**)

To visualize intrahepatic lymphatic vessels, antibodies targeting established lymphatic markers such as PROX-1 (Prospero homeobox protein 1), LYVE-1 (Lymphatic Vessel Endothelial Receptor 1) and Podoplanin were used. Unfortunately, none of the lymphatic marker antibodies showed specific staining. (**Supplementary Figure 1B** ) Further optimizing and validation of lymphatic marker antibodies will be pursued.

### Immunolabelling tumor cells using 3DISCO/iDISCO+

To investigate tissue clearing compatibility with immunolabeling of colorectal cancer tumor cell markers, we used a murine model in which colorectal cancer organoids were inoculated into the murine cecum. In this model, tumor cells spontaneously metastasize to the liver. (**Figure 1D**) Samples of tumor-bearing liver tissue were harvested. To identify a reliable tumor marker, antibodies against CD44 (a glycoprotein overexpressed in various cancers) and HES (mammalian Hairy enhance of split-1) were used. However, no specific staining was seen. (**Supplementary Figure 1C**) Using a tissue sample of the diaphragm with macroscopic tumor lesions, another possible tumor marker, EpCAM (Epithelial adhesion molecule), was evaluated. This tumor marker showed specific staining of the CRC tumor cells on the diaphragm tissue. (**Figure 3H**) Before usage in the clearing and immunolabelling protocol, EpCAM labelling was validated on murine colorectal liver metastases immunohistochemistry (IHC) sections. (**Figures 3D, G**)

## Discussion

In this study, we investigated methods for murine liver tissue clearing to visualize the intrahepatic microvasculature as well as tumor cells in murine colorectal metastases. The field of tissue clearing offers a wide variety of clearing techniques, each with their own advantages and drawbacks (the discussion of which is beyond the scope of this paper). Most tissue clearing research has focused on neurobiology as well as made use of endogenous fluorescence while imaging. For the aim of this study to visualize intrahepatic microvasculature and tumor cells, a clearing technique was needed that could 1) adequately render liver tissue transparent (hampered by the liver’s high heme content) and 2) was compatible with immunolabelling vasculature and CRC tumor cells.

At the time, CLARITY and especially 3DISCO/iDISCO+ had shown immunolabelling compatibility for many antibodies in the neurobiology field, as well as proven capable of liver clearing. Therefore, for this study these clearing techniques were chosen. Unfortunately, in this study CLARITY using passive clearing did not allow suitable immunolabelling due to tissue integrity difficulties. While CLARITY is designed to embed samples into a polymer scaffold, hereby providing support to better preserve tissue structure (5, 18), as passive clearing of liver lobes took several weeks, the extended chemical exposure damaged the hydrogel-supported tissue structure. The active clearing CLARITY approach which is used to great effect in many studies (2, 3, 18, 22, 23), uses an electrochemical gradient which results in fast clearing. However, this requires specific equipment that was not available for us at this time. Molina et al. (24) showed an adjusted version of active CLARITY with immunolabelling to visualise liver bile ducts. Therefore, active CLARITY techniques with liver-focused antibody labelling could still provide an interesting clearing technique for imaging the liver (tumor) micro-environment.

Most optical clearing methods aim to preserve endogenous fluorescent proteins and counteract fluorescence quenching. The 3DISCO method created by Ertürk et al. (19) was further adapted by Renier et al. (20) to especially focus on whole-mount immunolabeling and imaging of large-volume samples. This method, dubbed iDISCO (immunolabeling-enabled 3D imaging of solvent-cleared organs), was then further adjusted by Chédotal and colleagues (3, 21). In this study, the 3DISCO/iDISCO+ approach successfully cleared liver tissue while maintaining tissue integrity and allowed immunolabeling of intrahepatic vasculature and CRC tumor cell markers (MECA-32 and EpCAM, respectively).

Currently, more clearing techniques are developed or adjusted to suit other organ systems besides the neurobiology field and better suit immunolabelling. Therefore, in the future more immunolabelling clearing options will be available for tumor micro-environment imaging in various organ systems. However, for specifically investigating the spatial organization of such tissues, maintaining tissue integrity is crucial. In many clearing protocols, such as iDISCO+, the solvents result in the expansion or shrinkage of the tissue samples (1). While some studies report shrinkage in all dimensions hereby maintaining similar spatial distribution, others suggest different amounts of shrinkage for different tissues, which could warp the original tissue and organ structure (19, 25, 26). On the other hand, tissue shrinkage can also benefit imaging depth as it renders larger samples smaller. In future studies, the influence of solvents on tissue dimensions and integrity should be considered when studying tissue spatial organization. Further investigation is needed to elucidate the reaction of different tissue components to the clearing solvents.

To our knowledge, only three other studies attempted to visualize both the liver’s endothelial and lymphatic structures. Oren et al. (23) made use of transgenic mice with a tdTomato fluorescent marker under the Vecad promotor to visualize blood vessels. In this study LYVE-1 was used as a lymphatic endothelial cell marker, however, it is not specific for liver lymphatic vessels as the sinusoidal endothelial cells also express LYVE-1 (27). Bobe et al. (28), in addition to LYVE-1 used co-stainings with PROX-1, VEGFR-3, and podoplanin for visualizing intrahepatic lymphatic embryonic development. Future studies will need to determine whether this method can be used to study the lymphatic vessels of the intrahepatic tumor micro-environment. Messal et al. (29) developed the FLASH method which was used to clear and label many organ tissues, such as the liver. In liver tissue, they labelled structures as bile ducts, smooth muscle cell markers for vasculature as well as PROX-1 for lymphatic vasculature. Moreover, they imaged *in vitro* tumor organoids in matrigel as well as pancreatic cancer tissue. It would be interesting to use this method for further imaging of the liver tumor-microenvironment, especially using other lymphatic markers such as podoplanin.

Validation of antibodies is a vital step of clearing methods. As tissue clearing protocols make use of harsh chemicals, such as methanol, many antibodies are not compatible. While time consuming and often costly, to be able to specifically label proteins of interest, an evaluation of various antibodies, different dilutions and incubation times is needed for immunolabelling optimization (1, 2, 4). Various tissue clearing methods have dedicated online databases documenting which antibodies were tested. As we currently used smaller samples for establishing this method, further optimization will be needed when we broaden the method to whole liver imaging. One of the major challenges is adequate antibody penetration into whole-organ samples. Recently, to improve antibody penetration the use of high-pressure delivery systems for antibodies as well as the use of nanobodies have been reported (6).

In this study, the approach to immunolabel the intrahepatic blood vasculature was chosen. To visualize blood vessels, intravenous injection of fluorescent agents such as fluorescently-labelled lectin can be used (15, 22). As this offers a strong fluorescent signal in for instance the 488 nm laser range, it might offer an alternative to blood vessel staining within a laser range that is often excluded due to strong autofluorescence. Moreover, many research groups visualize their protein of interest using transgenic fluorescent reporter mice. However, this offers a new set of challenges concerning creating transgenic mice and the quenching of endogenous fluorescence during the harsh chemical process of tissue clearing. Recently, Stamatelos et al. (30) and Ehling et al. (31) used micro-CT imaging to study tumor microvasculature, blood flow, and angiogenesis in murine xenografts. Moreover, Bhargava et al. (32) developed a comprehensive approach named VascuViz combining micro-MRI and micro-CT with possible optical clearing and imaging to visualize whole-organ and tumor microvasculature. To image blood vasculature, fluorescent CT and MRI contrast agents were combined for intracardial perfusion, after which tissue samples were collected, fixed and imaged by micro-MRI and micro-CT. Then tissue samples were used for IHC immunolabelling or for tissue clearing and imaging of endogenous fluorescent proteins and the fluorescent contrast agents. Afterwards, all data was integrated. This technique would require access to micro-MRI and micro-CT equipment as well as data integration expertise. In future studies, it would be fascinating to use this VascuViz technique to combine micro-MRI and micro-CT imaging with immunolabelling of various tumor micro-environment cell types such as cancer cells, immune cells and cancer-associated fibroblasts.

Whole organ clearing has also been used to investigate cancer metastasis evolution in a variety of murine tumor models (22, 33–35). Regarding post-mortem human tumor tissue, small sections used for tissue clearing have been performed on breast neoplasms (34, 36), prostate cancer (37), microvasculature in colorectal cancer (38), as well as lymphatic vasculature of bladder cancers (39). Tanaka et al. used tissue clearing to study patterns of cancer heterogeneity in both a murine bladder model as well as in biopsy samples of human ovarian, pancreas, colon, head and neck, and lung cancers (40). Three-dimensional tumor micro-environment tissue clearing would be valuable as it would allow visualization of the spatial heterogeneity and interactions of tumor cells and other components such as immune cells, endothelial cells or cancer-associated fibroblasts (CAFs) (14, 36, 41–43). Where the question can be posed whether 2D immunohistochemistry can truly adequate and representatively capture tumor heterogeneity, whole-organ tissue clearing could offer a more comprehensive approach. In fact, Cuccarese et al. studied the spatial distribution of tumor- associated macrophages in murine pulmonary carcinoma and their response to drug delivery (44). In future studies, we expect the use of tissue clearing in evaluating drug penetration, distribution, accumulation, tissue retention and any therapeutic treatment effect to intensify (2, 42, 45, 46). Interestingly, whereas most clearing protocols target proteins of interest, recently combining whole-mount *in situ* hybridization techniques such as smFISH techniques with tissue clearing have been attempted successfully (47–49). While this development is very recent and needs further enhancement, it offers the interesting possibility of studying both proteins and RNA simultaneously in the 3D tumor micro-environment structure.

In conclusion, we used the 3DISCO/iDISCO+ method to successfully render murine liver tissue optically transparent and immunolabel intrahepatic vasculature using MECA-32 as well as colorectal tumor cells using EpCAM. Further optimization of immunolabelling intrahepatic lymphatic vasculature is needed. Moreover, the immunolabeling of tumor cells, which was successful in a tumor lesion on the diaphragm, will need to be optimized for liver tissue. Imaging intrahepatic blood and lymphatic vasculature as well as tumor cells would be an especially valuable technique for allowing visualization of the spatial heterogeneity and complex interactions of tumor cells and their environment in future studies.

## Data availability statement

The raw data supporting the conclusions of this article will be made available by the authors, without undue reservation.

## Ethics statement

The animal study was reviewed and approved by the National Competent Authority (Licence number AVD115002016614) and Animal Ethics Committee (UMC Utrecht).

## Author contributions

NF, SP, OK, IR, JH were involved in study concept and design. NF, LB, SP, JW, LW in acquisition of data. NF, SP, OK, JH in analysis and interpretation of data. NF, LB, JH in drafting of the manuscript. IR, OK, JH in critical revision of the manuscript. NF, SP OK, JH in statistical analysis. JH, OK, IR obtained funding. All authors contributed to the article and approved the submitted version.

## Supplementary material

Supplementary Figure 1 (A) Immunolabelling intrahepatic vasculature with CD31 (red) showed no specific staining. DAPI (blue) Scale bar = 100mm (B) Lymphatic endothelial markers PROX-1 (nuclear staining, green), LYVE-1 (green) and Podoplanin (green) showed no specific staining. DAPI (red). Scale bar = 100mm (C) Moreover, tumor markers CD44 and HES (both in turquoise) also showed no specific staining for labelling colorectal cancer cells. DAPI (red), CRC tumor liver metastasis (*). Scale bar = 100mm.

## References

[1] Gómez-Gaviro MV Sanderson D Ripoll J Desco M. Biomedical applications of tissue clearing and three-dimensional imaging in health and disease. iScience (2020) 23(8):101432. doi: 10.1016/j.isci.2020.101432 32805648 PMC7452225

[2] Tian T Yang Z Li X. Tissue clearing technique: Recent progress and biomedical applications. J Anat (2021) 238:489–507. doi: 10.1111/joa.13309 32939792 PMC7812135

[3] Vigouroux RJ Belle M Chédotal A. Neuroscience in the third dimension: shedding new light on the brain with tissue clearing. (2017) 10(1):33. doi: 10.1186/s13041-017-0314-y PMC5520295 28728585

[4] Susaki EA Ueda HR. Whole-body and whole-organ clearing and imaging techniques with single-cell resolution: Toward organism-level systems biology in mammals. Cell Chem Biol (2016) 23:137–57. doi: 10.1016/j.chembiol.2015.11.009 26933741

[5] Seo J Choe M Kim SY. Clearing and labeling techniques for large-scale biological tissues. Molecules Cells (2016) 39:439–46. doi: 10.14348/molcells.2016.0088 PMC4916395 27239813

[6] Ueda HR Ertürk A Chung K Gradinaru V Chédotal A Tomancak P. Tissue clearing and its applications in neuroscience. Nat Rev Neurosci (2020) 21:61–79. doi: 10.1038/s41583-019-0250-1 31896771 PMC8121164

[7] Spalteholz W. Uüber das durchsichtigmachen von menschlichen und tierischen Praüparaten und seine theoretischen bedingungen, nebst anhang: Uüber Knochenfaürbung. Leipzig: S. Hirzel (1911).

[8] Spalteholz W. Uüber das durchsichtigmachen von menschlichen und tierischen Praüparaten und seine theoretischen bedingungen, nebst anhang: Uüber Knochenfaürbung. Leipzig: S. Hirzel (1914).

[9] Susaki EA Tainaka K Perrin D Yukinaga H Kuno A Ueda HR. Advanced CUBIC protocols for whole-brain and whole-body clearing and imaging. Nat Protoc (2015) 10(11):1709–27. doi: 10.1038/nprot.2015.085 26448360

[10] Ren Z Wu Y Wang Z Hu Y Lu J Liu J. CUBIC-plus: An optimized method for rapid tissue clearing and decolorization. Biochem Biophys Res Commun (2021) 568:116–23. doi: 10.1016/j.bbrc.2021.06.075 34217010

[11] Chen L Li G Li Y Li Y Zhu H Tang L. UbasM: An effective balanced optical clearing method for intact biomedical imaging. Sci Rep (2017) 7(1):12218. doi: 10.1038/s41598-017-12484-3 28939860 PMC5610269

[12] Tainaka K Kubota SI Suyama TQ Susaki EA Perrin D Ukai-Tadenuma M. Whole-body imaging with single-cell resolution by tissue decolorization. Cell (2014) 159(4):911–24. doi: 10.1016/j.cell.2014.10.034 25417165

[13] Jing D Zhang S Luo W Gao X Men Y Ma C. Tissue clearing of both hard and soft tissue organs with the pegasos method. Cell Res (2018) 28(8):803–18. doi: 10.1038/s41422-018-0049-z PMC6082844 29844583

[14] Hofmann J Gadjalova I Mishra R Ruland J. Efficient tissue clearing and multi- organ volumetric imaging enable quantitative visualization of sparse immune cell populations during in fl ammation. Front Immunol (2021) 11:1–17. doi: 10.3389/fimmu.2020.599495 PMC7869862 33569052

[15] Feuchtinger A Walch A Dobosz M. Deep tissue imaging: a review from a preclinical cancer research perspective. Histochem Cell Biol (2016) 146:781–806. doi: 10.1007/s00418-016-1495-7 27704211

[16] Azaripour A Lagerweij T Scharfbillig C Jadczak AE Willershausen B Van Noorden CJ. A survey of clearing techniques for 3D imaging of tissues with special reference to connective tissue. Prog Histochem Cytochem (2016) 51(2):9–23. doi: 10.1016/j.proghi.2016.04.001 27142295

[17] Chanrion M Kuperstein I Barrière C El Marjou F Cohen D Vignjevic D. Concomitant notch activation and p53 deletion trigger epithelial-to-mesenchymal transition and metastasis in mouse gut. Nat Commun (2014) 5:5005. doi: 10.1038/ncomms6005 25295490 PMC4214431

[18] Tomer R Ye L Hsueh B Deisseroth K. Advanced CLARITY for rapid and high-resolution imaging of intact tissues. Nat Protoc (2014) 9:1682–97. doi: 10.1038/nprot.2014.123 PMC4096681 24945384

[19] Ertürk A Becker K Jährling N Mauch CP Hojer CD Egen JG. Three-dimensional imaging of solvent-cleared organs using 3DISCO. Nat Protoc (2012) 7(11):1983–95. doi: 10.1038/nprot.2012.119 23060243

[20] Renier N Wu Z Simon DJ Yang J Ariel P Tessier-Lavigne M. iDISCO : A simple, rapid method to immunolabel Large tissue samples for volume imaging. Cell (2014) 159(4):896–910. doi: 10.1016/j.cell.2014.10.010 25417164

[21] Belle M Godefroy D Couly G Malone SA Collier F Giacobini P. Tridimensional visualization and analysis of early human development resource tridimensional visualization and analysis of early human development. (2017) 169(1):161–73.e12. doi: 10.1016/j.cell.2017.03.008 28340341

[22] Lagerweij T Dusoswa SA Negrean A Hendrikx EML de Vries HE Kole J. Optical clearing and fluorescence deep-tissue imaging for 3D quantitative analysis of the brain tumor microenvironment. Angiogenesis (2017) 20(4):533–46. doi: 10.1007/s10456-017-9565-6 PMC5660146 28699046

[23] Oren R Fellus-Alyagor L Addadi Y Bochner F Gutman H Blumenreich S. Whole organ blood and lymphatic vessels imaging (WOBLI). Sci Rep (2018) 8(1):1–9. doi: 10.1038/s41598-018-19663-w 29362484 PMC5780490

[24] Molina LM Krutsenko Y Jenkins NEC Smith MC Tao J Wheeler TB. LiverClear: A versatile protocol for mouse liver tissue clearing. STAR Protoc (2022) 3(1):101178. doi: 10.1016/j.xpro.2022.101178 35243370 PMC8857608

[25] Pan C Cai R Quacquarelli FP Ghasemigharagoz A Lourbopoulos A Matryba P. Shrinkage-mediated imaging of entire organs and organisms using uDISCO. Nat Methods (2016) 13(10):859–67. doi: 10.1038/nmeth.3964 27548807

[26] Vulders RCM van Hoogenhuizen RC van der Giessen E van der Zaag PJ. Clearing-induced tisssue shrinkage: A novel observation of a thickness size effect. PloS One (2021) 16:e0261417. doi: 10.1371/journal.pone.0261417 34914768 PMC8675714

[27] Mouta Carreira C Nasser SM di Tomaso E Padera TP Boucher Y Tomarev SI. LYVE-1 is not restricted to the lymph vessels: Expression in normal liver blood sinusoids and down-regulation in human liver cancer and cirrhosis. Cancer Res (2001) 61(22):8079–84. 11719431

[28] Bobe S Beckmann D Klump DM Dierkes C Kirschnick N Redder E. Volumetric imaging reveals VEGF-c-dependent formation of hepatic lymph vessels in mice. Front Cell Dev Biol (2022) 10:949896. doi: 10.3389/fcell.2022.949896 36051444 PMC9424489

[29] Messal HA Almagro J Zaw Thin M Tedeschi A Ciccarelli A Blackie L. Antigen retrieval and clearing for whole-organ immunofluorescence by FLASH. Nat Protoc (2021) 16(1):239–62. doi: 10.1038/s41596-020-00414-z 33247285

[30] Stamatelos SK Bhargava A Kim E Popel AS Pathak AP. Tumor ensemble-based modeling and visualization of emergent angiogenic heterogeneity in breast cancer. Sci Rep (2019) 9:5276. doi: 10.1038/s41598-019-40888-w 30918274 PMC6437174

[31] Ehling J Theek B Gremse F Baetke S Möckel D Maynard J. Micro-CT imaging of tumor angiogenesis: quantitative measures describing micromorphology and vascularization. Am J Pathol (2014) 184(2):431–41. doi: 10.1016/j.ajpath.2013.10.014 PMC3920056 24262753

[32] Bhargava A Monteagudo B Kushwaha P Senarathna J Ren Y Riddle RC. VascuViz: a multimodality and multiscale imaging and visualization pipeline for vascular systems biology. Nat Methods (2022) 19(2):242–54. doi: 10.1038/s41592-021-01363-5 PMC8842955 35145319

[33] Kubota SI Takahashi K Nishida J Morishita Y Ehata S Tainaka K. Whole-body profiling of cancer metastasis with single-cell resolution. Cell Rep (2017) 20(1):236–50. doi: 10.1016/j.celrep.2017.06.010 28683317

[34] Rios AC Capaldo BD Vaillant F Pal B van Ineveld R Dawson CA. Intraclonal plasticity in mammary tumors revealed article intraclonal plasticity in mammary tumors revealed through Large-scale single-cell resolution 3D imaging. (2019) 35(4):618–32.e6. doi: 10.1016/j.ccell.2019.02.010 30930118

[35] Lin PY Peng SJ Shen CN Pasricha PJ Tang SC. PanIN-associated pericyte, glial, and islet remodeling in mice revealed by 3D pancreatic duct lesion histology. Am J Physiol - Gastrointest Liver Physiol (2016) 311:G412–22. doi: 10.1152/ajpgi.00071.2016 PMC6347070 27340125

[36] Sabdyusheva Litschauer I Becker K Saghafi S Ballke S Bollwein C Foroughipour M. 3D histopathology of human tumours by fast clearing and ultramicroscopy. Sci Rep (2020) 10(1):17619. doi: 10.1038/s41598-020-71737-w 33077794 PMC7572501

[37] van Royen ME Verhoef EI Kweldam CF van Cappellen WA Kremers GJ Houtsmuller AB. Three-dimensional microscopic analysis of clinical prostate specimens. Histopathology (2016) 69(6):985–92. doi: 10.1111/his.13022 27353346

[38] Liu YA Pan ST Hou YC Shen MY Peng SJ. 3-d visualization and quantitation of microvessels in transparent human colorectal carcinoma. (2013) 8(11):e81857. doi: 10.1371/journal.pone.0081857 PMC3843693 24324559

[39] Tanaka N Kaczynska D Kanatani S Sahlgren C Mitura P Stepulak A. Mapping of the three-dimensional lymphatic microvasculature in bladder tumours using light-sheet microscopy. Br J Cancer (2018) 118(7):995–9. doi: 10.1038/s41416-018-0016-y PMC5931093 29515257

[40] Tanaka N Kanatani S Tomer R Sahlgren C Kronqvist P Kaczynska D. Whole-tissue biopsy phenotyping of three-dimensional tumours reveals patterns of cancer heterogeneity. Nat Biomed Eng (2017) 1(10):796–806. doi: 10.1038/s41551-017-0139-0 31015588

[41] Hofmann J Keppler SJ. Tissue clearing and 3D imaging – putting immune cells into context. J Cell Sci (2021) 134(15):jcs258494. doi: 10.1242/jcs.258494 34342351

[42] Lee SS Bindokas VP Kron SJ Biology C Facility LM. Multiplex three-dimensional mapping of macromolecular drug distribution in the tumor microenvironment. Mol Cancer Ther (2019) 18:213–26. doi: 10.1158/1535-7163.MCT-18-0554 PMC6318001 30322947

[43] Almagro J Messal HA Zaw Thin M van Rheenen J Behrens A. Tissue clearing to examine tumour complexity in three dimensions. Nat Rev Cancer (2021) 21:718–30. doi: 10.1038/s41568-021-00382-w 34331034

[44] Cuccarese MF Dubach JM Pfirschke C Engblom C Garris C Miller MA. Heterogeneity of macrophage infiltration and therapeutic response in lung carcinoma revealed by 3D organ imaging. Nat Commun (2017) 8:14293. doi: 10.1038/ncomms14293 28176769 PMC5309815

[45] Dobosz M Ntziachristos V Scheuer W Strobel S. Multispectral fluorescence Ultramicroscopy : Three- dimensional visualization and automatic quantification of tumor morphology, drug penetration, and antiangiogenic treatment response. Neoplasia (2014) 16:1–13. doi: 10.1593/neo.131848 24563615 PMC3924547

[46] Kingston BR Muhammad A Ngai J Sindhwani S. Assessing micrometastases as a target for nanoparticles using 3D microscopy and machine learning. Proc Natl Acad Sci USA (2019) 116:14937–46. doi: 10.1073/pnas.1907646116 PMC6660785 31285340

[47] Sylwestrak EL Rajasethupathy P Wright MA Jaffe A Deisseroth K. Multiplexed intact-tissue transcriptional analysis at cellular resolution. Cell (2016) 164:792–804. doi: 10.1016/j.cell.2016.01.038 26871636 PMC4775740

[48] Yang B Treweek JB Kulkarni RP Deverman BE Chen CK Lubeck E. Single-cell phenotyping within transparent intact tissue through whole-body clearing. Cell (2014) 158(4):945–58. doi: 10.1016/j.cell.2014.07.017 PMC4153367 25088144

[49] Shah S Lubeck E Schwarzkopf M He TF Greenbaum A Sohn CH. Single-molecule RNA detection at depth by hybridization chain reaction and tissue hydrogel embedding and clearing. Development (2016) 143:2862–7. doi: 10.1242/dev.138560 PMC5004914 27342713

