# Supplementary figures and images for: Tissue clearing and immunostaining to visualize the spatial organization of vasculature and tumor cells in mouse liver
Source: Front Oncol. 2023 Mar 28;13:1062926. doi: 10.3389/fonc.2023.1062926 (PMC10108913; doi:10.3389/fonc.2023.1062926)

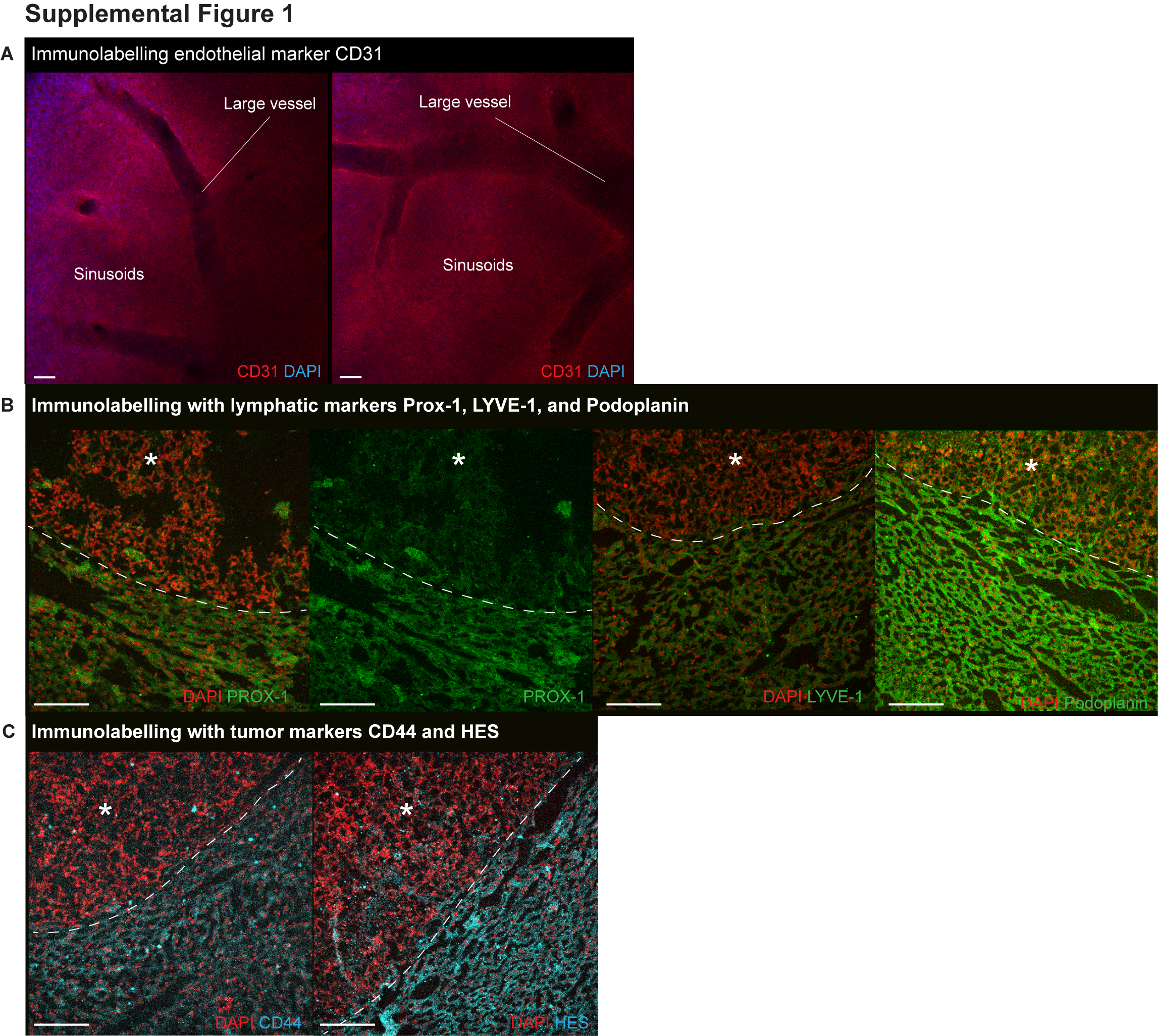

Supplement: Supplementary Figure 1 — (A) Immunolabelling intrahepatic vasculature with CD31 (red) showed no specific staining. DAPI (blue) Scale bar = 100mm (B) Lymphatic endothelial markers PROX-1 (nuclear staining, green), LYVE-1 (green) and Podoplanin (green) showed no specific staining. DAPI (red). Scale bar = 100mm (C) Moreover, tumor markers CD44 and HES (both in turquoise) also showed no specific staining for labelling colorectal cancer cells. DAPI (red), CRC tumor liver metastasis (*). Scale bar = 100mm. [file Image_1.tif]
